# Expression of Six1 in luminal breast cancers predicts poor prognosis and promotes increases in tumor initiating cells by activation of extracellular signal-regulated kinase and transforming growth factor-beta signaling pathways

**DOI:** 10.1186/bcr3219

**Published:** 2012-07-05

**Authors:** Ritsuko Iwanaga, Chu-An Wang, Douglas S Micalizzi, J Chuck Harrell, Paul Jedlicka, Carol A Sartorius, Peter Kabos, Susan M Farabaugh, Andrew P Bradford, Heide L Ford

**Affiliations:** 1 Department of Obstetrics and Gynecology, University of Colorado School of Medicine, 12700 East 19th Ave, Aurora, CO 80238 USA; 2 Program in Molecular Biology, University of Colorado School of Medicine, 12800 East 19th Ave., Aurora, CO 80238 USA; 3 Medical Scientist Training Program, University of Colorado School of Medicine, 12800 East 19th Ave., Aurora, CO 80238 USA; 4 Lineberger Comprehensive Cancer Center, University of North Carolina at Chapel Hill, 450 West Drive, Campus Box 7295, Chapel Hill, NC 27599 USA; 5 Department of Pathology, University of Colorado School of Medicine, 12800 East 19th Ave., Aurora, CO 80238 USA; 6 Department of Medicine, University of Colorado School of Medicine, 12800 East 19th Ave., Aurora, CO 80238 USA; 7 Department of Pharmacology, University of Colorado School of Medicine, 12800 East 19th Ave., Aurora, CO 80238 USA

## Abstract

**Introduction:**

Mammary-specific overexpression of Six1 in mice induces tumors that resemble human breast cancer, some having undergone epithelial to mesenchymal transition (EMT) and exhibiting stem/progenitor cell features. Six1 overexpression in human breast cancer cells promotes EMT and metastatic dissemination. We hypothesized that Six1 plays a role in the tumor initiating cell (TIC) population specifically in certain subtypes of breast cancer, and that by understanding its mechanism of action, we could potentially develop new means to target TICs.

**Methods:**

We examined gene expression datasets to determine the breast cancer subtypes with Six1 overexpression, and then examined its expression in the CD24^low^/CD44^+ ^putative TIC population in human luminal breast cancers xenografted through mice and in luminal breast cancer cell lines. Six1 overexpression, or knockdown, was performed in different systems to examine how Six1 levels affect TIC characteristics, using gene expression and flow cytometric analysis, tumorsphere assays, and *in vivo *TIC assays in immunocompromised and immune-competent mice. We examined the molecular pathways by which Six1 influences TICs using genetic/inhibitor approaches *in vitro *and *in vivo*. Finally, we examined the expression of Six1 and phosphorylated extracellular signal-regulated kinase (p-ERK) in human breast cancers.

**Results:**

High levels of Six1 are associated with adverse outcomes in luminal breast cancers, particularly the luminal B subtype. Six1 levels are enriched in the CD24^low^/CD44^+ ^TIC population in human luminal breast cancers xenografted through mice, and in tumorsphere cultures in MCF7 and T47D luminal breast cancer cells. When overexpressed in MCF7 cells, Six1expands the TIC population through activation of transforming growth factor-beta (TGF-β) and mitogen activated protein kinase (MEK)/ERK signaling. Inhibition of ERK signaling in MCF7-Six1 cells with MEK1/2 inhibitors, U0126 and AZD6244, restores the TIC population of luminal breast cancer cells back to that observed in control cells. Administration of AZD6244 dramatically inhibits tumor formation efficiency and metastasis in cells that express high levels of Six1 ectopically or endogenously. Finally, we demonstrate that Six1 significantly correlates with phosphorylated ERK in human breast cancers.

**Conclusions:**

Six1 plays an important role in the TIC population in luminal breast cancers and induces a TIC phenotype by enhancing both TGF-β and ERK signaling. MEK1/2 kinase inhibitors are potential candidates for targeting TICs in breast tumors.

## Introduction

*Six1 *is a homeodomain-containing transcription factor that belongs to the *Six *family of homeoproteins and is highly expressed in embryogenesis. The Six family members are known to play an important role in the expansion of precursor populations prior to differentiation [1-4]. In mice, absence of Six1 leads to the reduction in size or loss of multiple organs as a result of decreased proliferation and increased apoptosis [5-10]. Thus, inappropriate expression of the *Six *genes in adult tissue has the potential to contribute to tumor initiation. In support of this hypothesis, we have shown that aberrant expression of Six1 in adult mammary cells reinstates a pro-proliferative and pro-survival program that likely contributes to Six1-dependent transformation and tumor formation in xenograft and transgenic mouse models [11-13].

Six1 mRNA is overexpressed in 50% of primary breast cancers, and in a much larger 90% percent of metastatic lesions [14], suggesting that it may be involved in more than just tumor initiation. Indeed, our analysis of Six1 expression in several public microarray datasets from human breast cancers demonstrates that inappropriate overexpression of Six1 correlates significantly with worse survival [12]. We recently determined that, in addition to the role that Six1 plays in proliferation and survival, its overexpression also leads to the induction of an epithelial to mesenchymal transition (EMT) via upregulation of transforming growth factor-β (TGF-β) signaling. Since genes that induce EMT have been shown to increase the metastatic capability of cells [15,16], we previously investigated and demonstrated that Six1 overexpression in mammary carcinoma cells induces metastasis in both experimental and orthotopic mouse models of metastasis [12]. Interestingly, Six1 overexpression in the non-transformed mammary glands of transgenic mice leads to an increase in the mammary stem cell population, suggesting that Six1 may play a role in normal mammary stem cells [13]. Taken together, these data suggest that Six1 overexpression in mammary carcinoma cells may increase the cancer stem cell (CSC) or tumor initiating cell (TIC) population.

Herein we demonstrate for the first time that Six1 expression predicts poor prognosis, specifically in luminal subtypes of breast cancer where it is associated with the CSC population. Indeed, we show that Six1 can lead to the expansion of a luminal cancer stem-like cell, and that it does so via its ability to activate both the TGF-β signaling and mitogen activated protein kinase/extracellular signal-regulated kinase (MEK/ERK) signaling pathways. We further demonstrate that the MEK1/2 inhibitor, AZD6244, significantly reduces tumor initiating capability *in vivo *in breast cancer cells that ectopically and endogenously express high levels of Six1. Finally, we demonstrate that Six1 expression correlates with phosphorylated ERK (pERK) levels in human breast cancers, suggesting that Six1 mediates its tumor promotional activities through activation of both TGF-β (previously shown) [12] and MEK/ERK signaling in the human context. Taken together, our data present the novel finding that Six1 mediates an increase in the TIC population in luminal breast cancers via activating multiple signaling pathways.

## Materials and methods

### Cell culture

All cell lines were obtained from ATCC (American Type Culture Collection, Manassas VA, USA) and cultured per recommendations. Generation of MCF7-Ctrl, MCF7-Six1, and MCF7-Six1-TβRIIDN lines was described previously [12,17]. To tag the cells, one of three MCF7-Ctrl (B1) and MCF7-Six1 (A13) clones was transduced with pLNCX2-ZsGreen retrovirus and selected by fluorescence activated cell sorting (FACS). To generate 66cl4/Six1 KD cells, the cells were infected with a lentiviral vector encoding either a scramble control or an shRNA targeting Six1 (Open Biosystems, Lafayette CO, USA). Clonal isolates were chosen from the two most efficient knockdown clones, Six1 KD1 (5' AAACCCAGGGCTGCCTTGGAAAAG 3') and Six1 KD2 (5' AAACCCAGGGCTGCCTTGGAAAAG 3'), as assessed by examining both RNA and protein levels.

### Microarray analysis

Microarray analysis was previously performed as described [12]. The red, green and black color scale represents the expression level of a gene above, below and equal, respectively, to the mean expression of that probe across all samples. MCF7-Ctrl and MCF7-Six1 microarray data sets can be found in the NCBI GEO database. The accession number is GSE23655. All gene expression and clinical data from the 779 tumor dataset and UNC311 dataset is available [18] under the collection of publications: Harrell *et al.*, *Breast Cancer Research and Treatment *2012 and Prat *et al.*, *Breast Cancer Research *2010. Categorical survival analyses were performed using log-rank tests and visualized with Kaplan-Meier plots. Box-and-whisker plots show the relationship of the intrinsic subtypes with Six1 and were performed in R. Interquartile range (IQR) is shown by the colored box and the bar indicates the median value; whiskers are 1.5*IQR.

### Immunohistochemistry

Tumor arrays containing human breast invasive ductal cancer, with 71 cases/72 cores (US Biomax, BRC711, Rockville MD, USA) were treated as previously described [12,13]. The following primary antibodies were used: Six1 (1:100 Atlas Antibodies, Stockholm, Sweden) [12] and p-ERK (1:100 Phosphosolutions, Aurora CO, USA).

### Flow cytometry

Cultured cells or xenograft tumors were harvested and washed in 0.5% BSA-PBS after which 10^6 ^cells were stained in 20 ul of antibody on ice for 30 minutes. Cells were washed in 1 ml of 0.5% BSA-PBS and resuspended in 400 ul of 1 ug/ml DAPI/0.5% BSA-PBS after which flow cytometry was performed. The following antibodies were used; APC-linked anti human CD44 (1:1, #559942: BD Pharmingen, San Jose CA, USA), biotin-linked anti human CD24 (1:100, #13-0247-82; eBioscience, San Diego CA, USA), and PE-linked streptavidin (1:200, #554061; BD Biosciences-Pharmingen, San Jose CA, USA). Fluorescence was detected with CyAn (Beckman Coulter, Brea CA, USA).

### Tumorsphere assay

Tumorsphere assays were performed as described in Dontu *et al. *[19] with cells seeded at a density of 2000 cells/2 ml in 6-well dishes. For the single cell sphere assay, single cells from the primary tumorspheres (1 cell/200 μl/well) were plated out in 96-well ultra-low attachment plates (Costar, Austin TX, USA) and spheres counted at 10 to 14 days.

### Western blot analysis

Western blot analysis was performed on whole cell lysates prepared as previously described [20] or with nuclear extracts. The following primary antibodies were used: E-cadherin (1:2500, #610181, BD Biosciences, San Jose CA, USA), β-catenin (1:750, #610154, BD Biosciences, San Jose CA, USA), p-ERK (1:1000, #9101S, Cell Signaling, Danvers MA, USA), total ERK (1:1000, #9102, Cell Signaling, Danvers MA, USA), β-actin (1:5000, Sigma, St. Louis MO, USA), and Six1 (1;1000), which was made as previously described [20]. Quantitation was performed using the Quantity One version 4.6.2 software (Bio-Rad, Hercules CA, USA).

### Xenograft models

Breast tumors were collected after surgical resection at The University of Colorado Hospital. Female NOD-scid IL2Rg^null ^mice four- to seven-weeks old were purchased from Jackson Laboratories, Bar Harbor ME, USA. Solid pieces of primary tumors (10 mm^3^) were dipped into Matrigel (BD Biosciences) and inserted into the #4 mammary fat pads of anesthetized recipient mice using a 10-gauge trochar. The animals were implanted subcutaneously with single silastic pellets containing 17β-estradiol (2 mg). Tumors were removed at necropsy from animals when they reached 1 to 1.5 cm in diameter and were treated with 1 mg/ml collagenase IV (Sigma) at 37 degrees Celsius for one hour. Clinical descriptions of tumors were: PE 4; ER+(90%)PR+(75%)HER2-, PK12; ER+(93%)PR+(15%)HER2-, and PK15; ER+(8%)PR-HER2-. Studies were performed with Institutional Review Board approval and informed consent of all patients. All animal studies were performed under an institutional animal care and use committee (IACUC) approved protocol.

### Tumor formation assay

MCF7 cells or 66Cl4 cells serially diluted in 100 μl of 1:1 PBS/Matrigel (#354234, BD Biosciences) were injected underneath the nipple of the #4 mammary fat pad of six-week old female NOD/SCID or BALB/c mice. Tumor formation efficiency was monitored weekly by palpation. For AZD6244 treatment, 1 × 10^4 ^MCF7 cells were injected into the mammary fat pads of six-week old female NOD/SCID mice. One week post injection, mice were treated by oral gavage with 25 mg/kg or 50 mg/kg AZD6244 or vehicle (10% EtOh, 10% Cremophor EL, 80% D5W), twice per day for three days and once per day for the next three days. Animal studies were performed under an IACUC approved protocol. The statistical analysis was performed using Extreme Limiting Dilution Analysis [21].

### Metastasis assay

A total of 1 × 10^6 ^66cl4/scramble or 66cl4/Six1KD cells were suspended in 100 μl of (D)MEM and injected into the mammary fat pad of six-week old female Balb/C mice. One week post-injection, mice were treated with 50 mg/kg AZD6244 or vehicle by oral gavage, twice per day for seven days. Three weeks post cell injection, mice were injected with D-luciferin, and imaged using the IVIS200 imaging system. Quantitation of luciferase signal was performed by measuring flux in lungs and axillary lymph nodes of animals and using the LivingImage version 2.6 software.

## Results

### Six1 expression correlates with poor prognosis in luminal breast cancers, particularly the luminal B subtype

**Figure 1:**
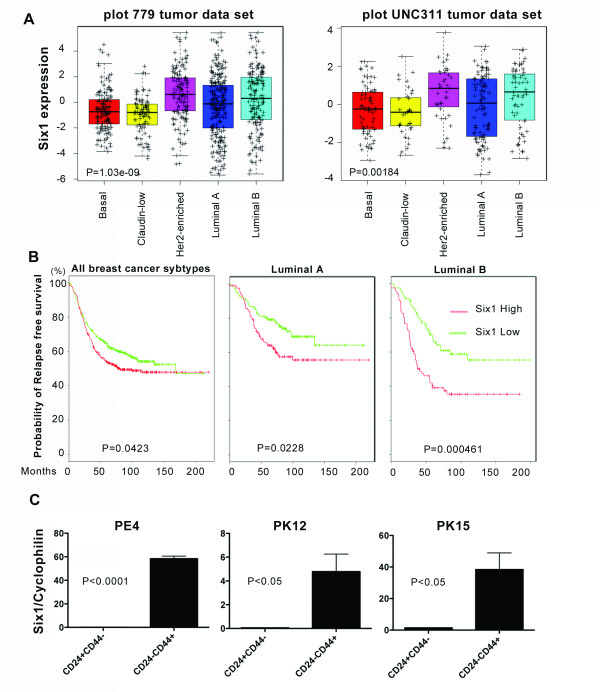
**Six1 expression correlates with poor prognosis in luminal breast cancer and is high in luminal breast cancer TICs**. (**A**) Association of Six1 with intrinsic subtype. Box-and-whisker plots are shown for two independent datasets, 779 tumor dataset and UNC311 dataset. *P *values were calculated with ANOVA. Individual *P*-values comparing expression of Six1 in luminal B tumors to other subtypes are as follows: 779 dataset versus Basal = 0.0004, Claudin = 1.43E-6, HER2 = 0.07, LumA = 0.03. UNC311 dataset versus Basal = 0.0089, Claudin = 0.0046, HER2 = 0.4561, LumA = 0.0388. (**B**) Relapse free survival curve of Kaplan-Meier analyses with a combined 779 breast tumor data set. Kaplan Meier curves are shown for all breast cancer subtypes, Luminal A tumors, and Luminal B tumors. (**C**) Six1 mRNA expression was determined by RT-PCR, and normalized to Cyclophilin B mRNA. Luminal B patient xenografts PE4, PK12 and PK15 were injected in NOD-scid IL2Rg^null ^mice, after which the tumors were excised, CD24^+^CD44^- ^and CD24^low ^CD44^+ ^populations isolated, and Six1 mRNA levels determined. PE4; ER+(90%)PR+(75%)HER2-, PT12; ER+(93%)PR+(15%)HER2-, and PT15; ER+(8%)PR-HER2-. ANOVA, analysis of variance; RT-PCR, reverse transcriptase-polymerase chain reaction.

Because Six1 expression induces an EMT both *in vitro *and *in vivo*, a phenotype that is primarily associated with basal and claudin low breast cancers, we examined whether its expression was enriched specifically in these subtypes of breast cancer using the previously combined 779 breast tumor dataset [22] and UNC311 dataset [23]. Both datasets include patients with early stage breast cancers as well as with locally advanced disease. While expression of Six1 could be found in all breast cancer subtypes, to our surprise, the highest levels of Six1 mRNA were found in human epidermal growth factor receptor 2 (Her2)-enriched and luminal B breast cancers (Figure 1A). Furthermore, within this large dataset, we found that Six1 correlates with shortened relapse free survival when examining all breast cancers (Figure 1B), but that this correlation is caused primarily by the effect of Six1 in the luminal breast cancer subtypes, particularly the luminal B subtype (Figure 1B). In fact, high expression of Six1 does not predict poor prognosis in other tumor subtypes [See Additional File 1, Figure S1A]. Importantly, when we performed a univariate analysis within 243 luminal A tumors and 162 luminal B tumors, Six1 expression and metastasis rate was significantly correlated only in the luminal B subtypes (*p *= 0.0177). These data suggest that, despite inducing an EMT-like phenotype, Six1 may, in fact, play a particularly important role in luminal B breast cancers, which are highly aggressive and refractory to tamoxifen therapies.

Because previous studies demonstrated a role for Six1 in EMT and in the expansion of the mammary stem cell populations [12,13], and because Six1 correlates with poor prognosis primarily in luminal breast cancers, we reasoned that Six1 may play an important role in the TIC population within this subtype of breast cancer. Thus, we examined the expression of Six1 in the putative TIC population from primary human luminal type breast cancers that had been xenografted through NOD-scid IL2Rg^null ^mice. Human luminal B breast cancer xenografts (profiled by J. C. Harrell to establish subtype) were excised from mice and dissociated using collagenase. Flow cytometry was then performed using the human TIC surface markers Lin-, CD24 and CD44 [24], which importantly have also been implicated in TIC characteristics in luminal cancers specifically [25]. Six1 expression was significantly elevated in the CD24^low^CD44^+ ^human TIC population when compared to the CD24^+^CD44^- ^non-stem cell population in the three different xenografted human tumors examined (Figure 1C, Additional File 1, Figure S1B).

To determine whether Six1 levels are higher in the TIC population of cultured luminal breast cancer cell lines, thus enabling their use for mechanistic studies, we performed the functional tumorsphere assay to enrich for TICs in MCF7 and T47D luminal breast cancer cells [26]. Similar to our observation in human breast cancers xenografted in mice, we detected significantly higher Six1 mRNA in secondary tumorspheres from MCF7 and T47D cells, as compared to their adherent counterparts [See Additional File 1, Figure S1C and D respectively].

### Six1 expression in MCF7 cells leads to differential regulation of genes found in the breast TIC-gene signature

**Figure 2:**
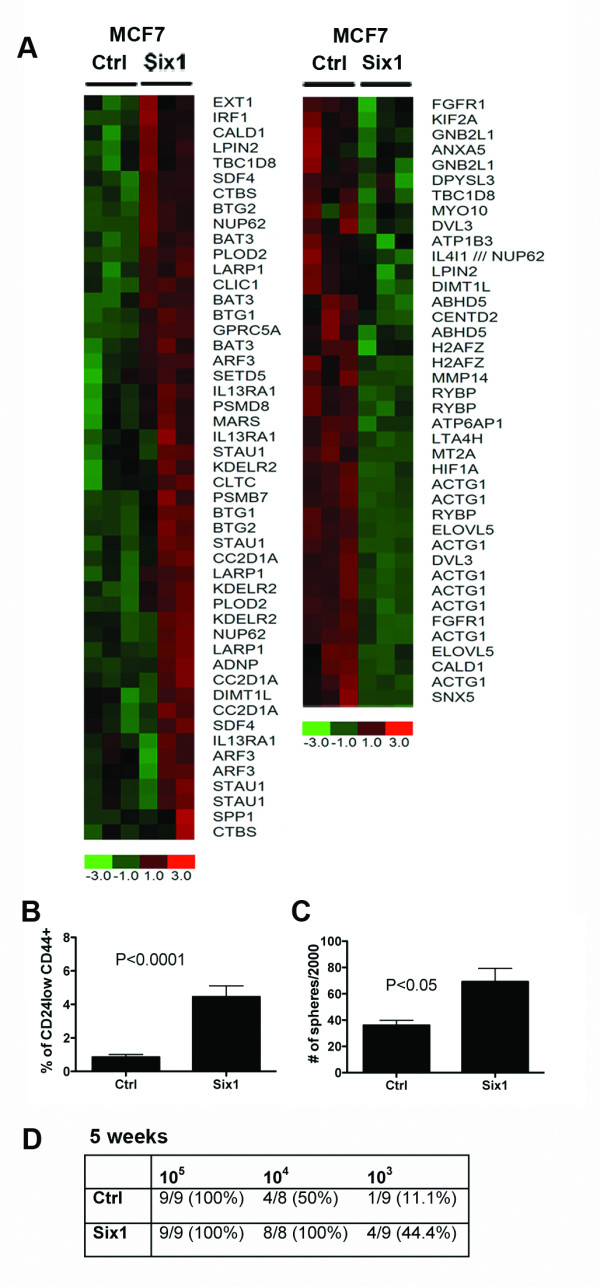
**Six1 overexpression leads to an increase in TICs**. (**A**) Six1 differentially regulates genes in the TIC signature. Microarray analysis was performed on MCF7-Six1 and Ctrl cells [17]. Expression data were filtered for probesets included in the CSC gene list from Shipitsin *et al. *[24] that had a 'present' call in at least 50% of the microarrays. Genes were clustered using hierarchical clustering. The color scale represents the expression level of a gene above (red), below (green), and at (black) the mean expression level of that gene across all samples. (**B**) Graph of the percent CD24^low ^CD44^+ ^cells found in MCF7-Six1 and MCF7-Ctrl cells. The graph represents an average of at least three independent experiments with three individual clones. Error bars represent mean +/- SEM. *P *values represent statistical analysis using a two-tailed *t *test. (**C**) Tumorsphere assays demonstrate that Six1 overexpression increases functional TICs. Secondary tumorsphere assays performed by plating cells from the primary tumorsphere in ultra low attachment plates and culturing for ten days. The graph represents three individual clones. Error bars represent mean value +/- SEM. *P *values represent statistical analysis using a two-tailed *t *test. The experiments were performed at least three times. (**D**) Six1 overexpression in MCF7 cells promotes tumor initiation in NOD/SCID mice. A total of 10^5 ^to 10^3 ^MCF7-Ctrl or MCF7-Six1 cells were injected into the #4 mammary fat pad of six-week old female NOD/SCID mice, and tumor formation monitored. Data shown from five weeks post injection. Statistical analysis performed using Extreme Limiting Dilution Analysis. Ctrl versus.. Six1; *P *< 0.001. CSC, cancer stem cell; Ctrl, control; SEM, standard error of the mean; TIC, tumor initiating cell.

Because Six1 expression is increased in TICs of both xenografted human luminal breast cancers and cell lines, we directly assessed whether Six1 overexpression could lead to an expansion of TICs in the MCF7 luminal mammary carcinoma cell line. Microarray analysis was performed on previously established MCF7 cell lines overexpressing Six1 (MCF7-Six1) versus control MCF7 cells (MCF7-Ctrl) [17] and the gene expression signatures were compared to human breast TIC signatures published by two independent groups [24,27] (Figure 2A and Additional File 2, Figure S2A). In both datasets, genes identified in the signature were differentially regulated in MCF7-Six1 cells when compared to MCF7-Ctrl cells. These data strongly suggest that Six1 alters the expression of genes associated with the TIC phenotype.

### Overexpression of Six1 increases the percentage of TICs in MCF7 cells

Since MCF7-Six1 cells display an altered TIC-like gene signature, we asked whether Six1 increases the overall percentage of TICs when overexpressed in MCF7 cells. To test this possibility, we compared the percentage of TICs between MCF7-Ctrl and MCF7-Six1 cells using flow cytometry after staining the cells with antibodies against CD24 and CD44 [28]. We found that MCF7-Six1 cells display a fivefold increase in the CD24^low^CD44^+ ^putative breast TICs relative to the MCF7-Ctrl cells (Figure 2B, Additional File 2, Figure S2B). To determine whether the increased CD24^low^CD44^+ ^population represents a functional increase in TICs, tumorsphere assays were performed. Secondary tumorsphere assays, which measure self-renewal capability, demonstrate that Six1 overexpression results in a two-fold increase in tumorsphere formation efficiency (Figure 2C). Because the tumorsphere assay may lead to aggregation, we additionally performed the assay after plating single cells per well in 96-well plates to assess TIC activity. As shown in Additional File 3, Figure S3A, secondary tumorsphere assays performed on single cells after sorting demonstrated that Six1 overexpression results in a 1.5-fold increase in the efficiency of formation of tumorspheres. It should be noted that the overall number of MCF7 cells that can form spheres in a single cell assay is significantly higher than that in a standard assay, perhaps because cell aggregation leads to an underestimate of sphere number in the standard sphere assay. Nonetheless, taken together these data strongly suggest that Six1 is able to increase the percentage of functional TICs when overexpressed in luminal type mammary carcinoma cells.

To determine conclusively whether Six1 overexpression augments the functional TIC compartment, we serially diluted MCF7-Six1 or MCF7-Ctrl cells (three individual clones of each line) and injected them orthotopically into NOD/SCID mice. Five weeks after orthotopic injection of 10^4 ^cells, MCF7-Six1 cells formed tumors 100% of the time, whereas MCF7-Ctrl cells formed tumors only 50% of the time. When the number of cells injected was reduced to 10^3^, 44% of the MCF7-Six1 formed tumors, whereas only 11% of the MCF7-Ctrl cells formed tumors (*P *< 0.001) (Figure 2D and Supplemental Figure S2C). Together, these data demonstrate that Six1 overexpression in luminal MCF7 breast cancer cells significantly increases the tumor initiating capability of these cells.

### Six1 expands the MCF7 TIC population through activating TGF-β signaling

**Figure 3:**
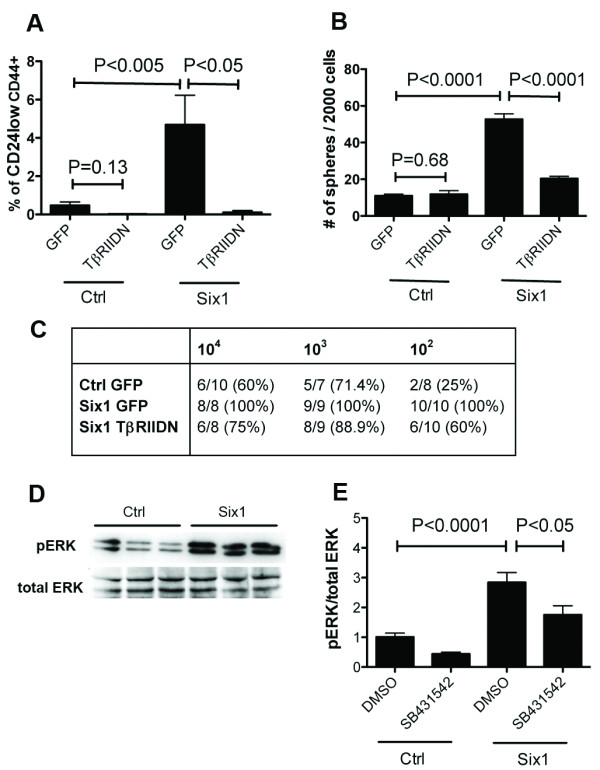
**Six1 is dependent on TGF-β and MEK/ERK signaling to increase TICs**. (**A**) Inhibition of TGF-β signaling leads to reduction of the CD24^low ^CD44^+ ^TICs in MCF7-Six1 cells. The graph represents an average of at least three experiments. Error bars represent mean +/**- **SEM. *P *values calculated with a two-tailed *t *test. (**B**) Inhibition of TGF-β signaling reverses Six1-dependent increases in tumorsphere formation. Representative secondary tumorsphere assay at d10. Experiments were performed at least three times. Error bars represent mean +/**- **SEM. *P *values calculated using a two-tailed *t *test. (**C**) Six1 expands the MCF7 TIC population through TGF-β signaling. Decreasing cell numbers were injected into the #4 mammary fat pad of six-week old female NOD/SCID mice, and the mice were monitored for tumor formation (five week data shown). Statistical analysis was performed using Extreme Limiting Dilution Analysis: Ctrl versus Six1, *P *< 5E-08; Ctrl versus TβRIIDN, *P *= 0.067; Six1 versus TβRIIDN, *P *< 5E-05. (**D**) Six1 activates ERK phosphorylation. Western blot analysis performed on lysates from three MCF7-Ctrl and MCF7-Six1 clones using anti-pERK (1:1000) and anti-total ERK (1:1000). (**E**) Six1 increases ERK phosphorylation in part through TGF-β signaling. The TGF-β type I receptor kinase inhibitor, SB431542, partially represses Six1-mediated ERK phosphorylation. MCF7-Ctrl and MCF7-Six1 cells (three clones each) were treated with 1 μM SB431542 or DMSO for 48 hours after which Western blot analysis was performed and analyzed with Quantity one software (v4.6.2 Bio-Rad). The graph is an average of at least three experiments with three clones. Error bars represent mean +**/- **SEM. *P *values calculated using a two-tailed *t *test. Ctrl, control; ERK, extracellular growth factor receptor; MEK, mitogen activated protein kinase; SEM, standard error of the mean; TGFβ, transforming growth factor β; TIC, tumor initiating cell.

We have shown that Six1 activates TGF-β signaling [12] and that the activation of TGF-β signaling by Six1 is required for its ability to induce EMT and metastasis [12]. Importantly, activation of TGF-β signaling induces TICs, providing a strong link between EMT, TICs, and metastatic disease [28-30]. To assess whether TGF-β signaling is required for the Six1-induced increase in TICs, we performed the tumorsphere assay on MCF7-Ctrl and MCF7-Six1 cells treated with SB431542, a TGF-β type I receptor kinase inhibitor. SB431542 treatment inhibited TGF-β signaling in both MCF7-Ctrl and MCF7-Six1 cells [See Additional File 4, Figure S4A]; however, tumorsphere formation efficiency was only inhibited in MCF7-Six1 cells, but not in MCF7-Ctrl cells [See Additional File 4, Figure S4B]. These data suggest that Six1-mediated upregulation of TGF-β signaling is required for the ability of Six1 to increase the functional TIC population, and that Six1-expressing cells are strongly dependent on this pathway for the induction of TICs. To eliminate the possibility that the SB431542 may diminish the Six1-induced TIC population via off target effects, we utilized a second system, in which MCF7-Six1 cells were stably transfected with a TGF-β Type II receptor dominant negative construct (TβRIIDN) [12]. MCF7-Six1/TβRIIDN cells were first examined to insure that TGF-β signaling was decreased in the presence of the TβRIIDN using a 3TP-luciferase reporter assay. As previously demonstrated [12], transcription from the Smad-responsive 3TP-luciferase construct is increased in MCF7-Six1 cells when compared to MCF7-Ctrl cells, and introduction of the TβRIIDN inhibits TGF-β signaling in both contexts [See Additional File 4, Figure S4C]. These cells were then used to examine the percentage of putative breast TICs (CD24^low^CD44^+^) in the absence or presence of Six1, and with or without active TGF-β signaling. MCF7-Six1/TβRIIDN cells contained a significantly lower percentage of CD24^low^CD44^+ ^cells when compared to the MCF7-Six1/GFP cells (Figure 3A). In contrast, inhibition of TGF-β signaling in MCF7-Ctrl cells did not significantly alter the percentage of putative TICs as measured by flow cytometry (Figure 3A). Furthermore, tumorsphere formation efficiency was also dramatically reduced when TGF-β signaling was inhibited in the MCF7-Six1 cells, but not in MCF7-Ctrl cells (Figure 3B). Together, these data demonstrate that Six1 overexpressing cells are uniquely sensitive to inhibition of TGF-β signaling, and that they depend on the TGF-β pathway to augment the TIC population.

### TGF-β signaling is partially required for Six1-induced tumor initiation *in vivo *

To confirm that the TGF-β pathway is required for the ability of Six1 to initiate tumors *in vivo*, we injected MCF7-Ctrl/GFP, MCF7-Six1/GFP, or MCF7-Six1/TβRIIDN cells at limiting dilutions into the mammary fat pads of NOD/SCID mice, as described above. As expected, the MCF7-Six1 cells were dramatically more efficient at inducing tumors than the MCF7-Ctrl cells, which in this experiment was most evident at 10^2 ^cells (*P *< 5E-08) (Figure 3C, and Additional File 4, Figure S4D). The greater efficiency of tumor formation in this experiment as compared to that shown in Figure 2D is likely due to the fact that one clonal isolate was used from MCF7-Ctrl and MCF7-Six1 cells, as opposed to three of each, since one isolate needed to be chosen to make the TβRIIDN cells. Interestingly, the MCF7-Six1/TβRIIDN cells formed tumors at an intermediate level between MCF7-Ctrl and MCF7-Six1 cells (Ctrl versus TβRIIDN, *P *= 0.067; Six1 versus TβRIIDN, *P *< 5E-05). These data suggest that the TGF-β pathway is a critical, but not the only pathway, required by Six1 to mediate tumor initiation *in vivo*. Tumor size was not significantly different between the MCF7-Six1/GFP and MCF7-Six1/TβRIIDN [See Additional File 4, Figure S4E], suggesting that the decrease in tumor initiation was not merely a consequence of decreased growth rates of the tumor cells. Upon re-examination of the tumorsphere data, an intermediate phenotype was also observed when comparing MCF7-Ctrl/GFP to MCF7-Six1/TβRIIDN (Figure 3B, P < 0.0001). Overall, these data strongly suggest that the Six1-induced increase in TICs is in part dependent on the TGF-β pathway, but that Six1 may affect other TIC-inducing pathways as well.

### Six1 increases the TIC population via activating the MEK/ERK signaling pathway

Since TGF-β signaling is likely not the only mechanism by which Six1 induces TICs, we examined whether Six1 induces other signaling pathways that may be linked to TICs. The Raf/MEK/ERK signaling pathway has been linked to metastasis [31], EMT [32], and to cancer stem cells/tumor initiating cells [33]. Therefore, western blot analysis was performed to examine phosphorylation of ERK, which is a measure of activated ERK, in MCF7-Ctrl and MCF7-Six1 cells. Interestingly, a clear induction of pERK was seen with Six1 overexpression (Figure 3D). Since MEK/ERK kinases are known to be downstream of TGF-β in the non-canonical pathway [34], we determined whether activation of ERK in the MCF7-Six1 cells is dependent on TGF-β signaling by treating the cells with SB431542, which is known not to target ERK signaling directly [35]. Addition of SB431542 partially diminished the Six1-induced increase in pERK, but did not bring it back down to control levels (Figure 3E and Additional File 4, Figure S4F). Additionally, SB431542 treatment of MCF7-Ctrl cells diminished pERK levels. Together, these data suggest that MCF7 cells are in part dependent on TGF-β signaling to induce ERK signaling, but that Six1 impinges on MEK/ERK signaling in a manner that is independent of TGF-β. Thus, the data demonstrate that Six1 activates the MEK/ERK pathway via multiple mechanisms.

### MEK/ERK signaling is required to mediate the Six1-induced increase in breast TICs

**Figure 4:**
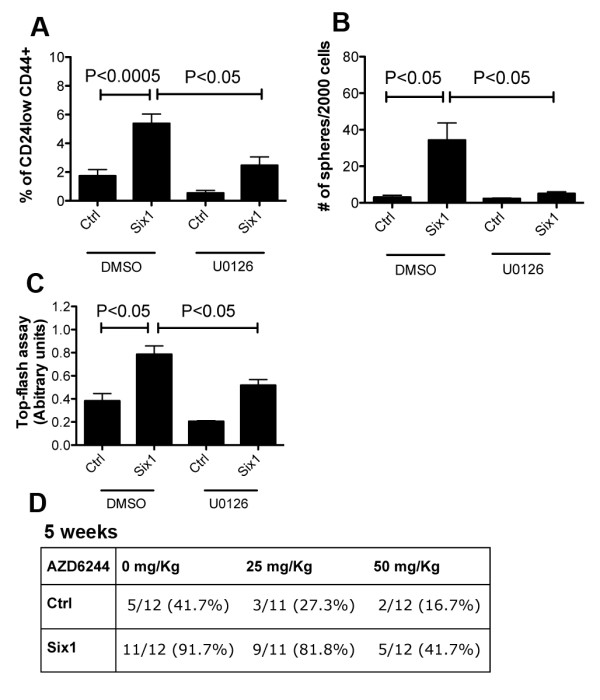
**Inhibition of MEK1/2 signaling attenuates Six1-induced increases in TICs**. (**A**) Inhibition of MEK1/2 with U0126 reduces the CD24^low ^CD44^+ ^TIC population. Cells were treated with10 μM U0126 for ten days. The graph represents an average of at least three experiments with three clones. Error bars represent mean +**/- **SEM. *P *values calculated using a two-tailed *t *test. (**B**) Inhibition of MEK1/2 with U0126 reduces secondary tumorsphere formation in MCF7-Six1 cells. Representative secondary tumorsphere assays at d10. The cells were treated with 10 μM U0126 throughout the assay. Experiments were performed at least three times. Error bars represent mean +/**- **SEM. *P *values calculated using a two-tailed *t *test. (**C**) Inhibition of MEK1/2 reverses β-catenin dependent transcription, as measured using a β-catenin responsive promoter (Top-flash) after normalization to renilla luciferase. Experiments were performed at least three times. Error bars represent mean +/**- **SEM. *P *values calculated using a two-tailed *t *test. (**D**) Inhibition of MEK1/2 by AZD6244 decreases tumor formation efficiency in NOD/SCID mice. A total of 10^4 ^MCF7-Six1 or MCF7-Ctrl cells (three clones each) were injected underneath the nipple of the fourth mammary gland of six-week old female NOD/SCID mice. One week post injection, the mice were treated with AZD6244 by oral gavage twice per day for three days and once/day for the following three days (vehicle, 25 mg/kg or 50 mg/kg) and were monitored weekly for tumor formation (five week data shown). Statistical analysis: Extreme Limiting Dilution Analysis. Six1 with vehicle versus Six1 with AZD6244 25 mg/kg, *P *< 0.05; Six1 with vehicle versus Six1 with AZD6244 50 mg/kg, *P *< 0.005. ERK, extracellular growth factor receptor; MEK, mitogen activated protein kinase; SEM, standard error of the mean; TIC, tumor initiating cell.

Since Six1 leads to an increase in ERK activation, we examined whether inhibition of MEK/ERK signaling, using the MEK1/2 kinase inhibitor U0126, decreases the ability of Six1 to enhance TICs. Western blot analysis was performed to examine phosphorylation of ERK and total ERK in lysates taken from MCF7-Ctrl and MCF7-Six1 cells treated with U0126 or with vehicle. U0126 inhibited phosphorylation of ERK both in MCF7-Ctrl and MCF7-Six1 cells [See Additional File 5 Figure S5A]. Flow cytometry assays to detect CD24^low^CD44^+ ^TICs in U0126 MCF7-Six1 treated cells as compared to vehicle treated cells showed a significant decrease in the TICs, bringing the percentage almost back down to that observed in MCF7-Ctrl cells (Figure 4A). In concert with the decrease in CD24^low^CD44^+ ^cells, tumorsphere formation efficiency was also decreased in MCF7-Six1 cells treated with U0126, to levels comparable to those observed in MCF7-Ctrl cells, suggesting that the MEK/ERK pathway is required for the ability of Six1 to increase the functional TIC population (Figure 4B). Because TICs and EMT go hand in hand [28], we asked whether MEK/ERK signaling may also impinge on the EMT induced by Six1 [12]. Indeed, U0126 treatment reversed the re-localization of E-cadherin and β-catenin observed in Six1 overexpressing cells, back to the levels in control cells [See Additional File 5, Figure S5B, C]. Moreover, inhibition of MEK/ERK with U0126 also reversed the ability of Six1 to induce transcriptional activation of the β-catenin TOP-FLASH reporter (Figure 4C). Overall, our data demonstrate that MEK/ERK signaling enhanced by Six1 is important for the induction of characteristics of EMT and TICs in MCF7 cells.

### Inhibition of MEK/ERK signaling decreases the tumor initiation capability of MCF7-Six1 cells

Because the commonly used MEK1/2 inhibitor, U0126, is not suitable for *in vivo *studies due to its associated toxicity [36], we instead used the highly specific MEK inhibitor, AZD6244, for studies performed in animals. AZD6244 does not perturb ATP-binding, but specifically blocks MEK activity [36]. It has been used in phase II clinical trials for patients with melanoma, non-small cell lung cancer, pancreatic cancer, breast cancer, colorectal cancer, as a single agent or in combination with other drugs. AZD6244 decreased secondary tumorsphere formation efficiency in MCF7-Six1 cells with equal potency to U0126 [See Additional File 5, Figure S5D, E]. When mice injected orthotopically with different concentrations of MCF7-Six1 cells were treated with AZD6244 (orally administered at 50 mg/kg/day or 100 mg/kg/day or vehicle), tumor initiation was significantly decreased up to five weeks post injection (vehicle versus AZD6244 25 mg/kg, *P *< 0.05; vehicle versus AZD6244 50 mg/kg, *P *< 0.005) (Figure 4D, Additional File 5, Figure S5F). However, treatment of MCF7-Ctrl injected mice with AZD6244 also significantly inhibited tumor initiation, suggesting that the MEK/ERK pathway is critical in tumor initiation in multiple contexts and that increased Six1 amplifies a pathway that is already required for tumor initiation. Regardless, inhibition of the MEK/ERK pathway may be a promising therapy to target TICs in luminal breast cancer. More importantly, these data suggest that targeting Six1 directly may also be an effective inhibitor of TICs as multiple pathways regulating the TIC phenotype including ERK and TGF-β pathways are activated by Six1.

### Endogenous Six1 regulates tumor initiation in an immunocompetent mouse model of breast cancer

**Figure 5:**
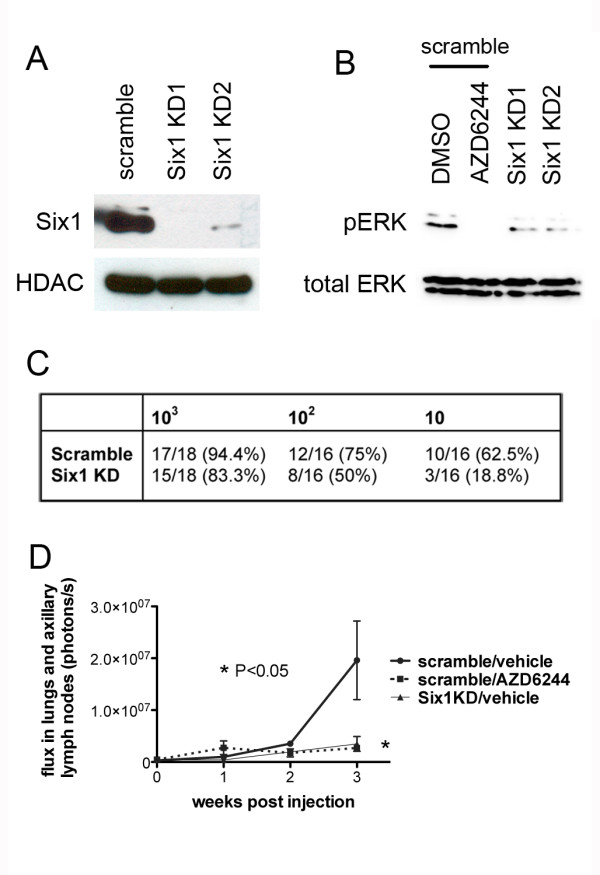
**Six1 is critical for ERK signaling and for tumor initiation in BALB/c mice**. (**A**) Western blot analysis demonstrates that introduction of a Six1 shRNA leads to an efficient decrease in Six1 protein in the cells. Nuclear extracts were isolated from 66cl4 control and Six1 knockdown cells and western blot analysis was performed with an anti-Six1 antibody (1:1000). An anti-HDAC antibody was used as a loading control. (**B**) AZD6244 treatment (second lane) and knockdown of Six1 (third and fourth lane) decreases pERK expression in 66cl4 cells. The cells were treated with 10 μM AZD6244 or vehicle for two hours. Whole cell lysates were used for Western blot analysis along with anti-pERK (1:1000) and anti-total ERK (1:1000) antibodies. (**C**) 66cl4 scramble control or Six1KD cells were serially diluted and then injected into the fourth mammary fat pad of six-week old female BALB/c mice. Mice were monitored for tumor initiation weekly. Data shown are from five weeks post injection of tumor cells. (**D**) A total of 10^6 ^66cl4/scramble or 66cl4/Six1KD cells were injected into the fourth mammary fat pad. One week post injection, mice were treated by oral gavage with vehicle or AZD6244 (50 mg^/^kg or vehicle twice per day for seven days). After injection of 150 mg of luciferin/kg into the mice, IVIS imaging was used to quantitate the metastastic burden every week. Error bars represent mean value +/- SEM. *P *values represent statistical analysis between 66cl4/scramble vehicle treated and AZD6244 treated mice, 66cl4/scramble vehicle treated and 66c14/Six1 KD vehicle treated mice, using a two-ta*i*led *t *test. ERK, extracellular signal-regulated kinase; SEM, standard error of the mean; shRNA, short hairpin RNA.

Although it is clear that Six1 overexpression leads to an increase in TICs, it is important to examine whether inhibition of Six1 could actually decrease the TIC population, thus affording a novel avenue by which TICs could be targeted, particularly in an immune-competent model. We thus performed shRNA-mediated knockdown of Six1 in the highly metastatic 66Cl4 mouse mammary carcinoma cell line (Figure 5A), which expresses high levels of endogenous Six1 and metastasizes from the orthotopic site when injected into syngeneic immunocompetent BALB/c mice [37]. Consistent with our results in MCF7 cells, we found that Six1 also modulates ERK signaling in this endogenous context, since knockdown (KD) of Six1 led to a decrease in pERK levels (Figure 5B).

To examine *in vivo *tumor formation efficiency in the context of Six1 KD, we performed the serial dilution/transplant assay using, in this case, an allograft model. Decreasing numbers of 66Cl4 scramble control KD cells (66cl4/scram), 66Cl4-Six1 KD1 and 66Cl4/Six1 KD2 cells were injected orthotopically into the mammary glands of BALB/c mice and tumor formation was monitored weekly. A significant decrease in tumor formation was observed with both Six1 KD cell lines, which was more pronounced at lower cell numbers (Figure 5C, Additional File 6, Figure S6A). Since Six1 is also important for cell cycle progression and the knock down of Six1 affects cell proliferation [38], we followed the experiment for eight weeks in the group of mice injected with 10^2 ^cells and 10 cells, and found that the tumor formation efficiency was not significantly altered from the five week time-point (not shown). These data suggest that the decrease in tumor initiation observed is not merely due to the difference in proliferation between 66Cl4 and 66Cl4/Six1KD, but may, at least in part, occur due to an alteration in of the number of TICs.

Because breast TICs are also associated with metastatic dissemination, we examined whether inhibition of the MEK1/2 kinase decreases not only tumor formation efficiency, but also metastasis. We thus performed an orthotopic metastasis assay as follows: 10^6 ^66cl4 cells were injected into the fourth mammary gland of BALB/c mice [37]. After one week, to allow the cells sufficient time to begin to form micrometastases in the lung [37], the mice received oral AZD6244 (or vehicle) two times daily at 50 mg/kg for seven additional days. The mice were imaged weekly using IVIS imaging. Intriguingly, even at three weeks post injection (and thus one week following cessation of treatment with AZD6244), the total metastatic burden (monitored by total flux in the lung and the axillary lymph nodes), was about five times less in AZD6244-treated relative to vehicle control treated animals (Figure 5D). Indeed, the decrease in metastatic burden (measured at three weeks) in response to MEK1/2 inhibition was similar to that observed with Six1 KD (Figure 5D). It should be noted that because the mice were treated with AZD6244 one week after cell injection, the effects of the drug could be on either metastatic dissemination and/or on metastatic outgrowth.

Importantly, in this experiment we also observed that AZD6244 treatment modestly decreased primary tumor size when compared to the control group, although this difference did not reach statistical significance, whereas the Six1 knockdown did reach statistical significance [See Additional File 6, Figure S6B]. Thus, it is possible that decreases in primary tumor burden influence the extent of metastasis both with MEK inhibition and Six1 inhibition, although Six1 inhibition has recently been shown to influence metastasis independent of primary tumor size [39]. Nonetheless, taken together, these data suggest that Six1 expression, and the MEK/ERK pathway, activated downstream of Six1, are important for tumor initiation, tumor burden, and subsequent metastasis in an allograft mammary tumor mouse model.

### pERK significantly correlates with Six1 expression in human breast cancer

**Figure 6:**
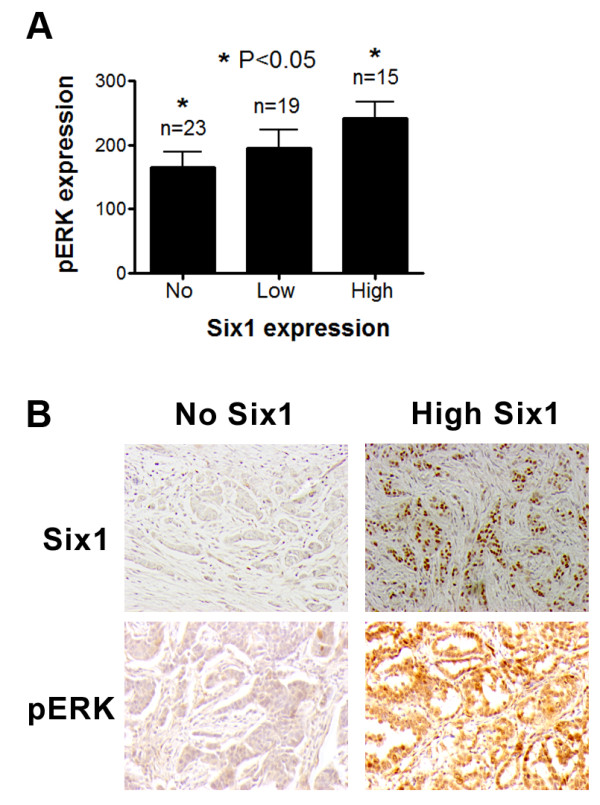
**pERK levels significantly correlate with Six1 expression in human breast cancer**. (**A**) Tumor arrays containing 72 human breast cancer samples were stained with anti-Six1 (1:100 *A*tlas antibodies) and anti-pERK (1:100 Phosphosolutions) antibodies. The immunostained arrays were scored in a blinded manner by a pathologist (PJ). Patients whose tumors have high levels of nuclear Six1, also have high levels of pERK, as assessed using the anti-Six1 antibody (nuclear Six1 intensity) and pERK expression (both nuclear and cytoplasmic, intensity times area). Error bars represent mean value +/**- **SEM. *P *values represent statistical analysis between no-Six1 expression and high-Six1 expression samples using a two-tailed *t *test. (**B**) Representative images of no Six1 and high Six1 expressing tumors, and of pERK staining in the same tumors. pERK, phosphorylated extracellular signal-regulated kinase; SEM, standard error of the mean.

Our results strongly suggest that Six1 increases TICs through activation of both TGF-β and MEK/ERK signaling in breast cancer cells. Indeed, we previously reported that Six1 and nuclear localization of the TGF-β effector Smad3 were significantly correlated in human breast cancer samples [12]. To examine whether ERK pathway activation also correlates with Six1 in human breast cancer, 72 human breast cancer tissues were stained with an anti-Six1 and anti-pERK antibody (Figure 6A, B). The expression levels of nuclear Six1 and levels of ERK phosphorylation (both nuclear and cytoplasmic pERK, intensity times percent of area) were significantly correlated (*P *< 0.05), These findings demonstrate that Six1 correlates with pERK in human breast cancer, and suggest that activation of ERK by Six1 may lead to expansion of TICs and to increased tumor aggressiveness.

## Discussion

In this paper we show that Six1 enhances a tumor initiating phenotype and that its expression is specifically associated with worsened prognosis in luminal B tumors. Within the paper, we use numerous means to conclusively demonstrate that Six1 induces a TIC phenotype through both TGF-β and ERK signaling, including examination of surface markers, tumorsphere assays, and *in vivo *tumor initiating assays. It should be noted that we have found that while Six1 enhances TICs as measured by *in vivo *tumor initiation in all contexts examined, we have found that changes in flow cytometric TIC markers are not always consistent with *in vivo *TIC results. These data suggest that the surface markers, while frequently used, are imperfect indicators of an *in vivo *tumor initiating phenotype, and that one should always use *in vitro *assays coupled with *in vivo *assays to make firm conclusions regarding TIC phenotypes.

Interestingly, while Six1 overexpressing luminal cells are uniquely dependent on TGF-β signaling to increase TIC populations *in vitro*, they are no more dependent than control cells on MEK/ERK signaling to induce some TIC characteristics *in vitro*, and for tumor initiation *in vivo*. Instead, Six1 overexpression increases the magnitude of MEK/ERK signaling. These data allow us to speculate that the MEK inhibitor, AZD6244, may be an attractive drug to target the luminal (and perhaps other) breast cancer TICs in any cells in which MEK/ERK signaling is active, but that Six1 overexpressing cells may require increased levels of the drug to accommodate the enhanced MEK/ERK signaling observed in those cells.

The mechanism by which Six1 activates MEK/ERK signaling is still unknown. It is known that TGF-β can activate the MEK/ERK pathway through a non-canonical pathway [32,34,40,41]. However, while our data indicate that Six1 may partially regulate MEK/ERK signaling downstream of TGF-β, it is not clear that this mechanism is solely responsible. Instead, we favor the hypothesis that Six1 regulates MEK/ERK signaling via TGF-β signaling as well as via regulating additional pathways, and that the induction of TGF-β signaling and MEK/ERK signaling together contribute to the ability of Six1 to induce TICs.

Both TGF-β signaling and MEK signaling have been implicated in EMT and TICs, and thus, Six1 upregulation of these pathways is consistent with the ability of Six1 to impart a TIC phenotype (Figure 5). Indeed, TGF-β signaling is an inducer of EMT and TICs in a variety of cells [12,24,28,42,43] and, in normal murine mammary gland epithelial cells, MEK/ERK signaling is required for TGF-β induced EMT [41]. MEK/ERK signaling has also been implicated in the induction of stem cell characteristics independent of TGF-β signaling. For example, inhibition of MEK/ERK signaling results in differentiation of human embryonic stem cells and human pluripotent stem cells into functional CD34+ progenitor cells [44], suggesting that MEK/ERK signaling is important for the maintenance of stem cell properties. Furthermore, MEK/ERK signaling has been implicated not only in normal stem cells, but in TICs [33].

Finally, our data demonstrate that Six1 expression in human tumors correlates both with activated TGF-β signaling and with activated ERK. It should be noted that the Six1 antibody used in these experiments was generated against a conserved region of Six1 and it may thus cross-react with other Six family members; therefore we can only confidently state that Six family member expression correlates with activated ERK. However, as Six1 is strongly correlated with prognosis in human breast cancers, and as its overexpression is observed in as many as 50% to 90% of breast cancers, it is likely that the staining is reflective of Six1 expression. In addition, we demonstrate that Six1 mRNA correlates with poor prognosis specifically in luminal type breast cancers. Taken together, these data suggest that combining ERK and TGF-β inhibitors may be an effective means of eliminating TICs in luminal type breast cancers, particularly in luminal B breast cancers.

## Conclusions

We show for the first time that Six1 expression correlates with poor prognosis in luminal breast cancers and, most significantly, in the aggressive luminal B subtype. We demonstrate that Six1 is overexpressed in the CD24^low^/CD44^+ ^TIC population from human luminal breast cancers, and that it can induce TICs when overexpressed in luminal breast cancer cells via its ability to activate both TGF-β and ERK signaling. We further show that endogenous Six1 can enhance tumor initiation in an immunocompetent mouse model, and in this context, where ERK signaling is regulated by Six1, inhibition of ERK signalling, dramatically decreases metastasis. Finally, we show for the first time that Six1 correlates with p-ERK in human breast tumors, suggesting that this mechanism is relevant to the human disease.

## Abbreviations

BSA: bovine serum albumin; Ctrl: Control; CSC: cancer stem cell; DAPI: 4',6-diamidino-2-phenylindole; (D)MEM: (Dulbecco's) modified eagle medium; EMT: epithelial to mesenchymal transition; ERK: extracellular signal-regulated kinase; EtOH: ethanol; FACS: fluorescence activated cell sorting; IACUC: institutional animal care and use committee; IQR: interquartile range; KD: knockdown; ER: estrogen receptor; MEK: mitogen activated protein kinase; NOD-SCID: non-obese diabetic/severe combined immunodeficiency; PBS: phosphate-buffered saline; PR: progesterone receptor; shRNA: short hairpin RNA; Six1: sine-oculis homeobox homolog 1; TβRIIDN: TGF-β Type II receptor dominant negative; TGF-β: transforming growth factor beta; TIC: tumor-initiating cell.

## Competing interests

The authors have applied for patents to target Six1 and to use Six1 as a marker, but these are not the subject of this manuscript and thus are not considered competing interests.

## Authors' contributions

RI conceived of and performed most of the experimentation throughout the manuscript, and wrote the initial draft of the manuscript. CW aided RI in performing experiments and interpreting results, and generated the Six1 knockdown cell lines used in the immunocompetent model. DSM, JCH, and SMF performed analysis of microarray datasets from MCF7 cells and of human breast cancers. PJ did all the mouse pathology, and scored the IHC on human sections. CS and PK xenografted the human luminal B tumors used in experiments and provided advice regarding experiments related to tumor initiating cells that were performed for Figure 1. AB contributed to the intellectual framework of the paper and provided feedback on experimentation and writing of the manuscript. HLF provided funding for the project, conceived of experiments along with RI, analyzed data along with RI, aided in writing of the manuscript along with RI. All authors have read and approved of the final manuscript.

## Supplementary Material

Additional file 1 **Figure S1**. **Six1 is enriched in the TIC population of luminal tumors**. (**A**) Relapse free survival curve of Kaplan-Meier analyses with a combined 779 tumor data set. Kaplan Meier curves are shown for Basal tumors, Claudin low tumors and Her2 positive tumors. (**B**) Flow cytometry was performed on the luminal B patient xenografts PK12, PK15 and PE4 following staining of the cells with antibodies that recognize CD24 and CD44. (**C **and **D**) The levels of Six1 mRNA are increased in MCF7 (B) and T47D (C) secondary tumorspheres as compared to the parental cells. Error bars represent mean value +/**- **SEM. *P *values represent statistical analysis using a two-tailed *t *test. The experiments were performed at least three times. Click here for file

Additional file 2 **Figure S2**. **Six1 overexpression enhances TIC characteristics**. (**A**) Six1 differentially regulates genes in the tumor initiating cell signature. The cancer stem cell/tumor initiating cell gene list was obtained from Liu *et al. *[45]. Microarray data from the MCF7-control and MCF7-Six1 expressing clones were filtered for genes included in the cancer stem cell/tumor initiating cell gene list and expression values across the samples were hierarchically clustered. The included gene expression data represents those genes that are consistently regulated across the clones. The color scale represents the expression level of genes above (red), below (green), and at (black) the mean expression level of that gene across all samples. (**B**) Six1 overexpression leads to an increase in tumor initiating cell characteristics. Flow cytometry was performed on MCF7-Six1 and MCF7-Ctrl cells following staining of the cells with antibodies that recognize CD24 and CD44. The boxed region represents the CD24^low ^CD44^+ ^TIC population. (**C**) Six1 over expression in MCF7 cells promotes tumor initiation in NOD/SCID mice. MCF7-Ctrl or MCF7-Six1 cells (10^5^, 10^4 ^and 10^3^) were injected into the number 4 mammary fat pad of six-week old female NOD/SCID mice, and the mice were then palpated weekly for tumor formation. Data shown are from two to four weeks post injection. Click here for file

Additional file 3 **Figure S3**. **Single cell tumorsphere assays demonstrate that Six1 overexpression increases the functional tumor initiating population**. (**A**) Single cell tumorsphere assays demonstrate that Six1 overexpression increases the functional tumor initiating population. Secondary tumorsphere assays were performed by plating single cells from the primary tumorsphere in 96-well ultra low attachment plates and culturing for ten days. The graph represents three individual clones. Error bars represent mean value +/- SEM. *P *values represent statistical analysis using a two-tailed *t *test. (**B**) Representative images of tumorspheres. Click here for file

Additional file 4 **Figure S4**. **TGF-β signaling is in part required for the ability of Six1 to induce TICs and pERK signalling**. (**A**) SB431542 inhibits TGF-β signaling in both MCF7-Ctrl and MCF7-Six1 cells, as assessed by TGF-β responsive promoter 3TP-luciferase activity after normalizing to renilla-luciferase. Error bars represent mean value **+/- **SEM. *P *values represent statistical analysis using a two-tailed *t *test. The experiments were performed at least three times. (**B**) Inhibition of TGF-β signaling by SB431542 reverses tumorsphere forming efficiency in MCF7-Six1 cells back to levels observed in the control cells. Representative secondary tumorsphere assay was shown after ten days of culture. The experiments were performed at least three times. Error bars represent mean value +/**- **SEM. *P *values represent statistical analysis using a two-tailed *t *test. (**C**) TβRIIDN inhibits TGF-β signaling in both MCF7-Ctrl and MCF7-Six1 cells, as assessed using the TGF-β responsive promoter 3TP-luciferase activity after normalizing to renilla-luciferase. Error bars represent mean value **+/- **SEM. *P *values represent statistical analysis using a two-tailed *t *test. The experiments were performed at least three times. (**D**) Six1 expands the MCF7 TIC population through activating TGF-β signaling. Decreasing numbers of MCF7-Six1, MCF7-Six1/TβRIIDN or MCF7-Ctrl cells were injected into the number 4 mammary fat pad of six-week old female NOD/SCID mice, and the mice were then palpated weekly for tumor formation. The data from two, three and four weeks post injection of tumor cells are shown. (**E**) Tumor volumes do not differ between MCF7-Six1/GFP and MCF7-Six1/TβRIIDN groups. Tumor volumes were measured five weeks post injection. The data shown are taken from tumors that formed in the group of mice injected with 10^3 ^cells. The tumor size was not significantly different between the MCF7-Six1/GFP and MCF7-Six1/TβRIIDN. *P *values represent statistical analysis using 1 way ANOVA. (**F**) The TGF-β type I receptor kinase inhibitor, SB431542, partially, but not completely, represses ERK phosphorylation activated by Six1. MCF7-Ctrl and MCF7-Six1 cells (three clones each) were treated with 1 μM SB431542 or vehicle (DMSO) for 48 hours after which whole cell lysates were collected and Western blot analysis was performed for p-ERK. The quantification is shown in Figure 3(E). Click here for file

Additional file 5 **Figure S5. MEK1/2 inhibitors inhibit EMT and TIC characteristics**. (**A**) The MEK1/2 inhibitor U0126 decreases pERK in MCF7-Six1 and MCF7-Ctrl cells. MCF7-Ctrl and MCF7-Six1 (three individual clones each) were treated with 10 μM U0126 or vehicle (DMSO) for two hours. The whole cell lysates were used to perform western blot analysis using anti-pERK (1:1000) and anti-total ERK (1:1000) antibodies. (**B **and **C**) U0126 inhibits the relocalization of E-cadherin (B) and β-catenin (C) observed with Six1 overexpression. Graphs represent quantitation of Western blots examining the soluble versus insoluble E-cadherin and β-catenin. The data represent mean value +/**- **SEM. *P *values represent statistical analysis using a two-tailed *t *test. (**D**) The MEK1/2 inhibitor AZD6244 decreases pERK in MCF7-Six1 and MCF7-Ctrl cells. MCF7-Ctrl and MCF7-Six1 (three individual clones each) cells were treated with 10 μM AZD6244 or vehicle (DMSO) for two hours. Whole cell lysates were extracted and used to perform Western blot analysis with anti-pERK (1:1000) and anti-total ERK (1:1000) antibodies. (**E**) Inhibition of MEK1/2 with AZD6244 dramatically reduces the secondary tumorsphere formation of MCF7-Six1 cells. Representative secondary tumorsphere assay is shown after ten days of culture. The experiments were performed at least three times. Error bars represent mean value +/**- **SEM. *P *values represent statistical analysis using a two-tailed *t *test. (**F**) Inhibition of MEK1/2 by AZD6244 dramatically decreases tumor formation efficiency in NOD/SCID mice. A total of 10^6 ^MCF7-Six1 or MCF7-Ctrl cells (three individual clones each) were injected underneath the nipple of the fourth mammary fat pad of six-week old female NOD/SCID mice. One week post injection, mice were treated with AZD6244 by oral gavage twice per day for three days and once per day for the following three days (vehicle, 50 mg/kg/day or 100 mg/kg/day). The mice were palpated weekly for tumor formation. The data from two, three and four weeks post injection of tumor cells are shown. Click here for file

Additional file 6 **Figure S6**. **Six1 KD inhibits both tumor initiation and metastasis in an immunocompetent model, and the MEK1/2 inhibitor, AZD6244, inhibits primary tumor burden similarly to Six1 KD**. (**A**) Knockdown of Six1 inhibits tumor initiation. 66cl4 scramble control or Six1KD cells were serially diluted and then injected into the fourth mammary fat pad of six-week old female BALB/c mice. Mice were monitored for tumor initiation weekly. The data from two, three and four weeks post injection of tumor cells are shown. (**B**) A total of 10^6 ^66cl4/scramble or 66cl4/Six1KD cells were injected into the fourth mammary fat pad. One week post injection, mice were treated by oral gavage with vehicle or AZD6244 at a concentration of 50 mg/kg or vehicle twice per day for seven days. After injection of 150 mg of luciferin/kg into the mice, IVIS imaging was used to quantitate the primary burden at three weeks post injection. Error bars represent mean value +/**- **SEM. It should be noted that the signal from a number (5/10) of the scramble control tumors was saturated at this time point, suggesting that the size of these tumors may be underestimated. In contrast, only 1/8 of 66cl4/scramble tumors in the AZD6244 group had saturated signal, and none of the tumors in the 66cl4/Six1 KD group had saturated signals. Click here for file
