# Supplementary figures and images for: Expression of Six1 in luminal breast cancers predicts poor prognosis and promotes increases in tumor initiating cells by activation of extracellular signal-regulated kinase and transforming growth factor-beta signaling pathways
Source: Breast Cancer Res. 2012 Jul 5;14(4):R100. doi: 10.1186/bcr3219 (PMC3680936; doi:10.1186/bcr3219)

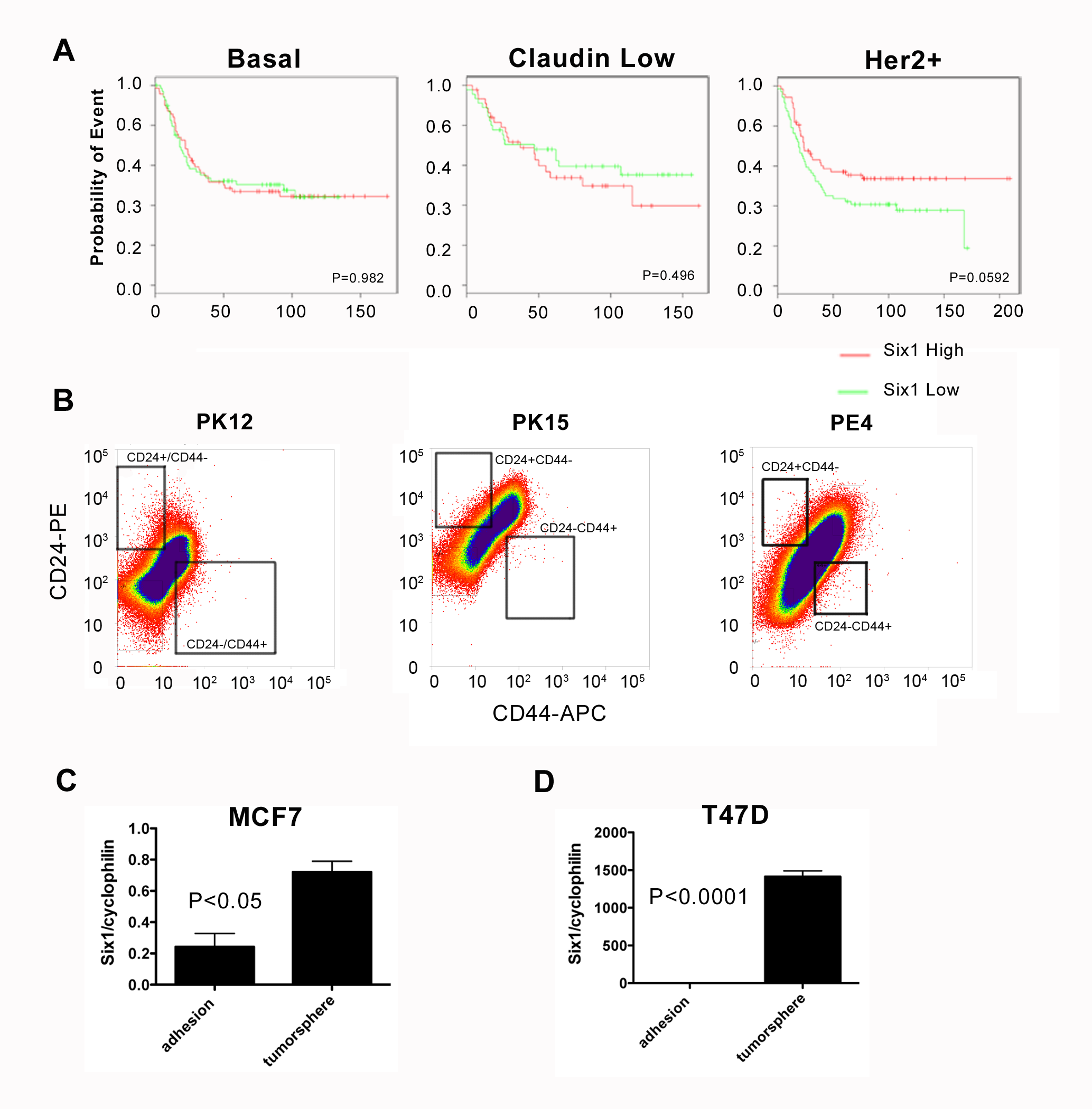

Supplement: Additional file 1 — Figure S1. Six1 is enriched in the TIC population of luminal tumors. (A) Relapse free survival curve of Kaplan-Meier analyses with a combined 779 tumor data set. Kaplan Meier curves are shown for Basal tumors, Claudin low tumors and Her2 positive tumors. (B) Flow cytometry was performed on the luminal B patient xenografts PK12, PK15 and PE4 following staining of the cells with antibodies that recognize CD24 and CD44. (C and D) The levels of Six1 mRNA are increased in MCF7 (B) and T47D (C) secondary tumorspheres as compared to the parental cells. Error bars represent mean value +/- SEM. P values represent statistical analysis using a two-tailed t test. The experiments were performed at least three times. [file bcr3219-S1.TIFF]

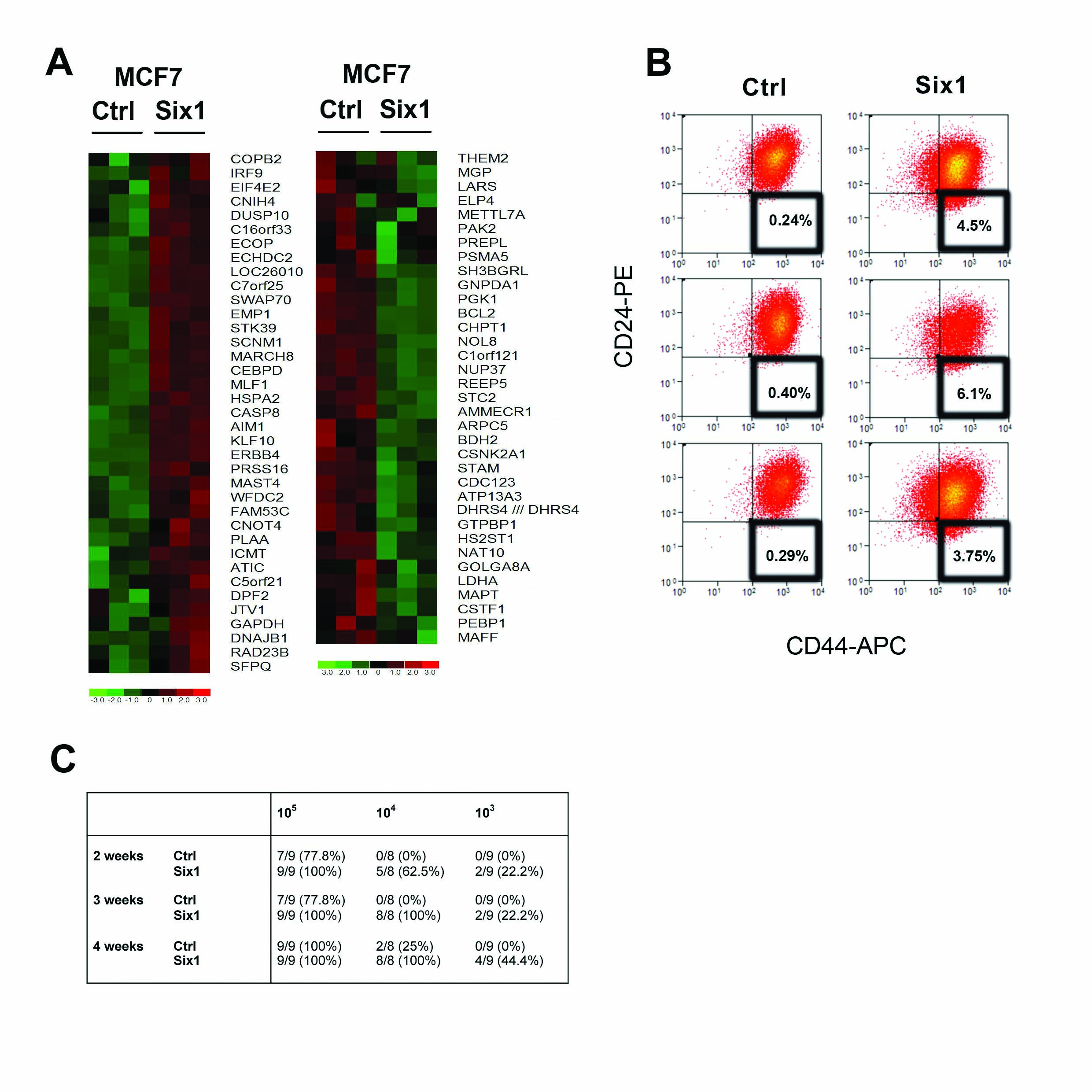

Supplement: Additional file 2 — Figure S2. Six1 overexpression enhances TIC characteristics. (A) Six1 differentially regulates genes in the tumor initiating cell signature. The cancer stem cell/tumor initiating cell gene list was obtained from Liu et al. [45]. Microarray data from the MCF7-control and MCF7-Six1 expressing clones were filtered for genes included in the cancer stem cell/tumor initiating cell gene list and expression values across the samples were hierarchically clustered. The included gene expression data represents those genes that are consistently regulated across the clones. The color scale represents the expression level of genes above (red), below (green), and at (black) the mean expression level of that gene across all samples. (B) Six1 overexpression leads to an increase in tumor initiating cell characteristics. Flow cytometry was performed on MCF7-Six1 and MCF7-Ctrl cells following staining of the cells with antibodies that recognize CD24 and CD44. The boxed region represents the CD24low CD44+ TIC population. (C) Six1 over expression in MCF7 cells promotes tumor initiation in NOD/SCID mice. MCF7-Ctrl or MCF7-Six1 cells (105, 104 and 103) were injected into the number 4 mammary fat pad of six-week old female NOD/SCID mice, and the mice were then palpated weekly for tumor formation. Data shown are from two to four weeks post injection. [file bcr3219-S2.JPEG]

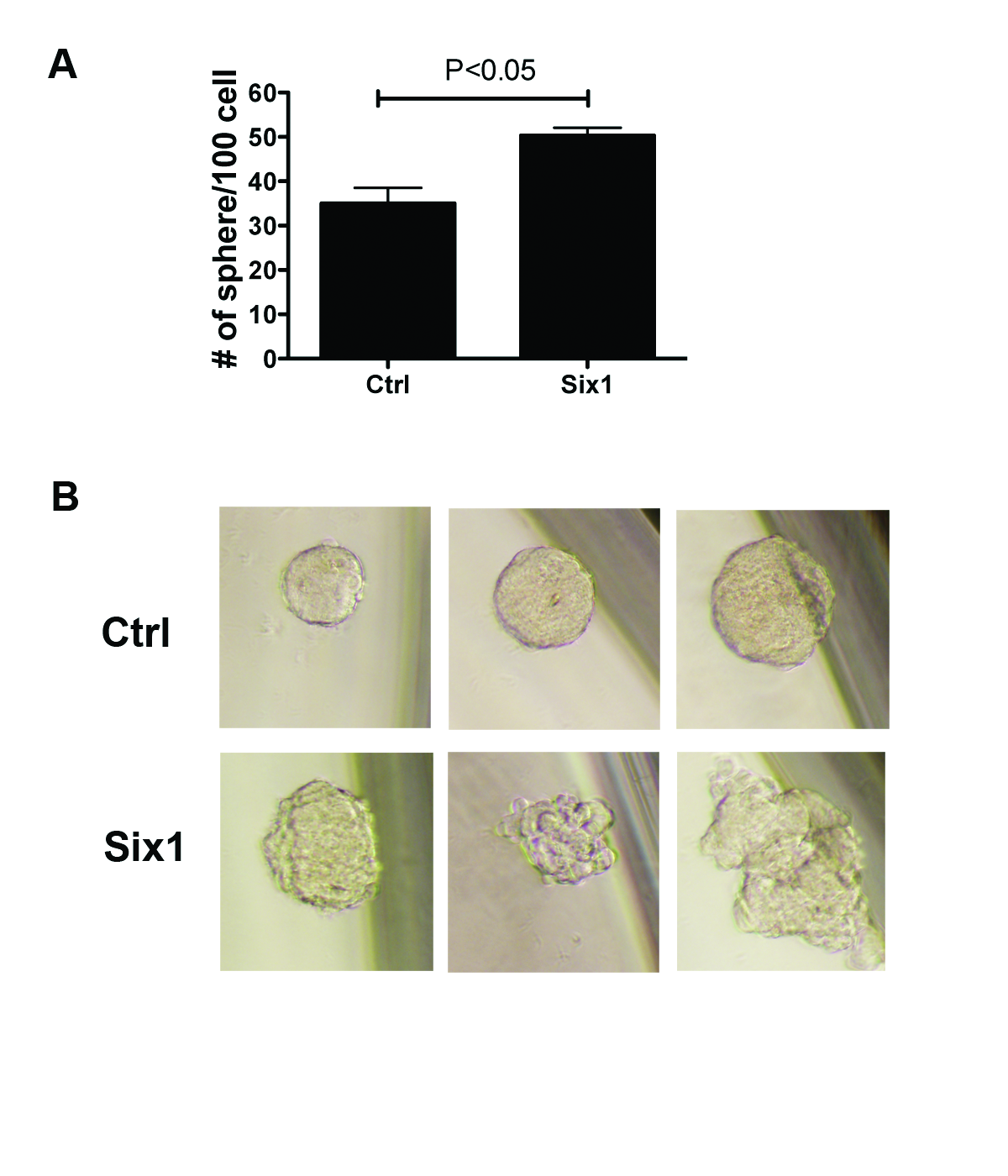

Supplement: Additional file 3 — Figure S3. Single cell tumorsphere assays demonstrate that Six1 overexpression increases the functional tumor initiating population. (A) Single cell tumorsphere assays demonstrate that Six1 overexpression increases the functional tumor initiating population. Secondary tumorsphere assays were performed by plating single cells from the primary tumorsphere in 96-well ultra low attachment plates and culturing for ten days. The graph represents three individual clones. Error bars represent mean value +/- SEM. P values represent statistical analysis using a two-tailed t test. (B) Representative images of tumorspheres. [file bcr3219-S3.TIFF]

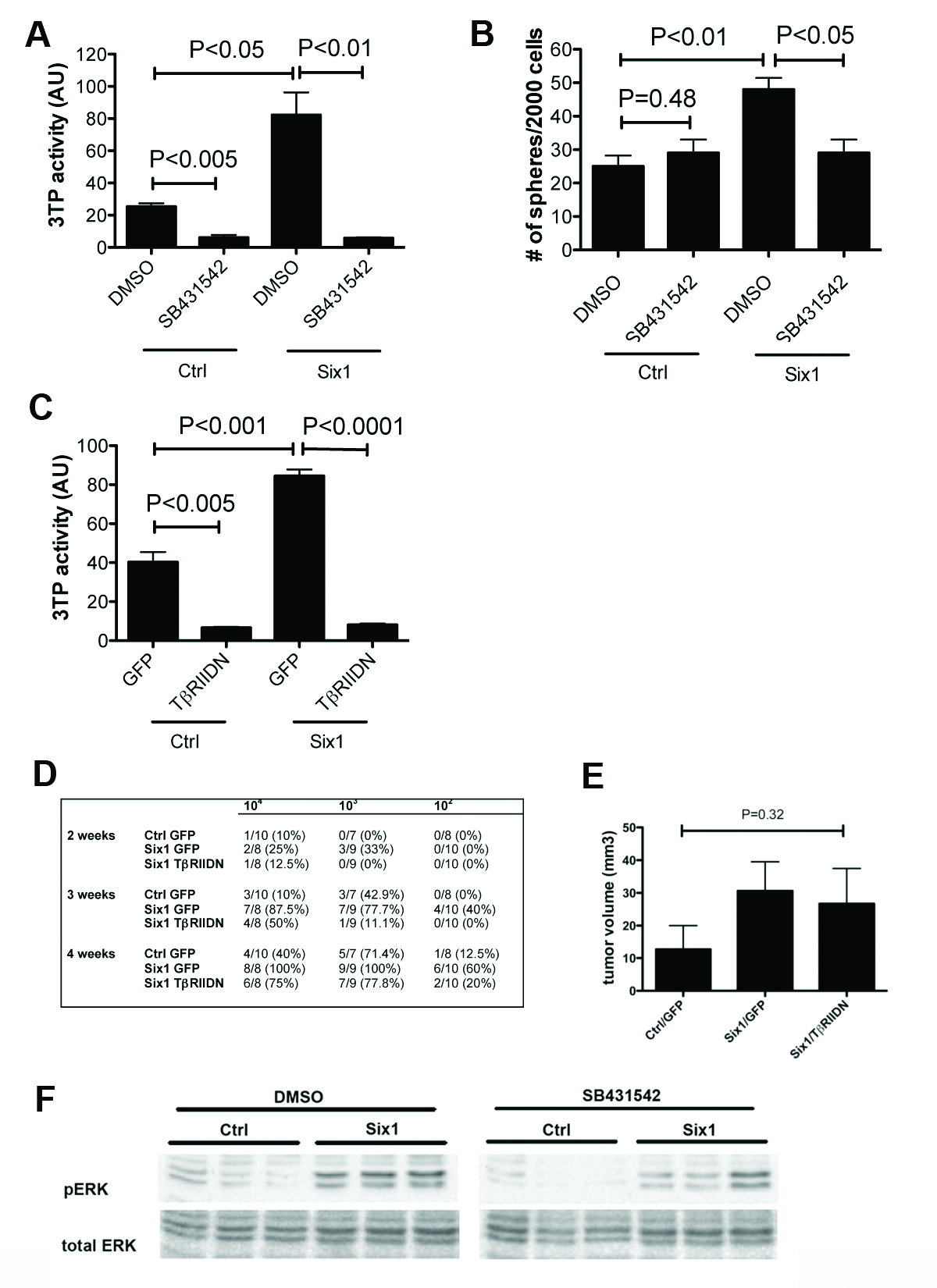

Supplement: Additional file 4 — Figure S4. TGF-β signaling is in part required for the ability of Six1 to induce TICs and pERK signalling. (A) SB431542 inhibits TGF-β signaling in both MCF7-Ctrl and MCF7-Six1 cells, as assessed by TGF-β responsive promoter 3TP-luciferase activity after normalizing to renilla-luciferase. Error bars represent mean value +/- SEM. P values represent statistical analysis using a two-tailed t test. The experiments were performed at least three times. (B) Inhibition of TGF-β signaling by SB431542 reverses tumorsphere forming efficiency in MCF7-Six1 cells back to levels observed in the control cells. Representative secondary tumorsphere assay was shown after ten days of culture. The experiments were performed at least three times. Error bars represent mean value +/- SEM. P values represent statistical analysis using a two-tailed t test. (C) TβRIIDN inhibits TGF-β signaling in both MCF7-Ctrl and MCF7-Six1 cells, as assessed using the TGF-β responsive promoter 3TP-luciferase activity after normalizing to renilla-luciferase. Error bars represent mean value +/- SEM. P values represent statistical analysis using a two-tailed t test. The experiments were performed at least three times. (D) Six1 expands the MCF7 TIC population through activating TGF-β signaling. Decreasing numbers of MCF7-Six1, MCF7-Six1/TβRIIDN or MCF7-Ctrl cells were injected into the number 4 mammary fat pad of six-week old female NOD/SCID mice, and the mice were then palpated weekly for tumor formation. The data from two, three and four weeks post injection of tumor cells are shown. (E) Tumor volumes do not differ between MCF7-Six1/GFP and MCF7-Six1/TβRIIDN groups. Tumor volumes were measured five weeks post injection. The data shown are taken from tumors that formed in the group of mice injected with 103 cells. The tumor size was not significantly different between the MCF7-Six1/GFP and MCF7-Six1/TβRIIDN. P values represent statistical analysis using 1 way ANOVA. (F) The TGF-β type I receptor kinase inhibit [file bcr3219-S4.TIFF]

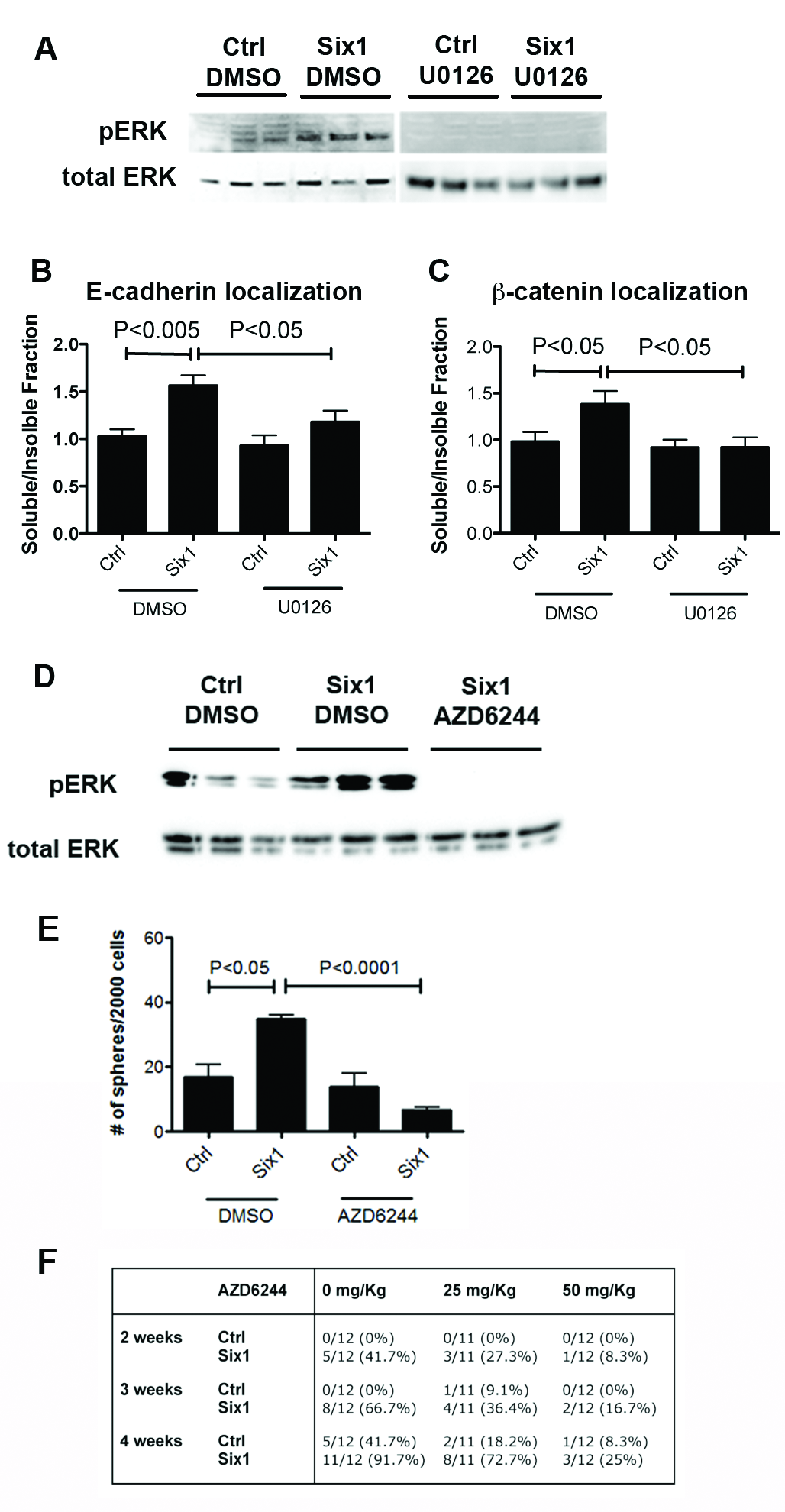

Supplement: Additional file 5 — Figure S5. MEK1/2 inhibitors inhibit EMT and TIC characteristics. (A) The MEK1/2 inhibitor U0126 decreases pERK in MCF7-Six1 and MCF7-Ctrl cells. MCF7-Ctrl and MCF7-Six1 (three individual clones each) were treated with 10 μM U0126 or vehicle (DMSO) for two hours. The whole cell lysates were used to perform western blot analysis using anti-pERK (1:1000) and anti-total ERK (1:1000) antibodies. (B and C) U0126 inhibits the relocalization of E-cadherin (B) and β-catenin (C) observed with Six1 overexpression. Graphs represent quantitation of Western blots examining the soluble versus insoluble E-cadherin and β-catenin. The data represent mean value +/- SEM. P values represent statistical analysis using a two-tailed t test. (D) The MEK1/2 inhibitor AZD6244 decreases pERK in MCF7-Six1 and MCF7-Ctrl cells. MCF7-Ctrl and MCF7-Six1 (three individual clones each) cells were treated with 10 μM AZD6244 or vehicle (DMSO) for two hours. Whole cell lysates were extracted and used to perform Western blot analysis with anti-pERK (1:1000) and anti-total ERK (1:1000) antibodies. (E) Inhibition of MEK1/2 with AZD6244 dramatically reduces the secondary tumorsphere formation of MCF7-Six1 cells. Representative secondary tumorsphere assay is shown after ten days of culture. The experiments were performed at least three times. Error bars represent mean value +/- SEM. P values represent statistical analysis using a two-tailed t test. (F) Inhibition of MEK1/2 by AZD6244 dramatically decreases tumor formation efficiency in NOD/SCID mice. A total of 106 MCF7-Six1 or MCF7-Ctrl cells (three individual clones each) were injected underneath the nipple of the fourth mammary fat pad of six-week old female NOD/SCID mice. One week post injection, mice were treated with AZD6244 by oral gavage twice per day for three days and once per day for the following three days (vehicle, 50 mg/kg/day or 100 mg/kg/day). The mice were palpated weekly for tumor formation. The data from two, three and four weeks post in [file bcr3219-S5.TIFF]

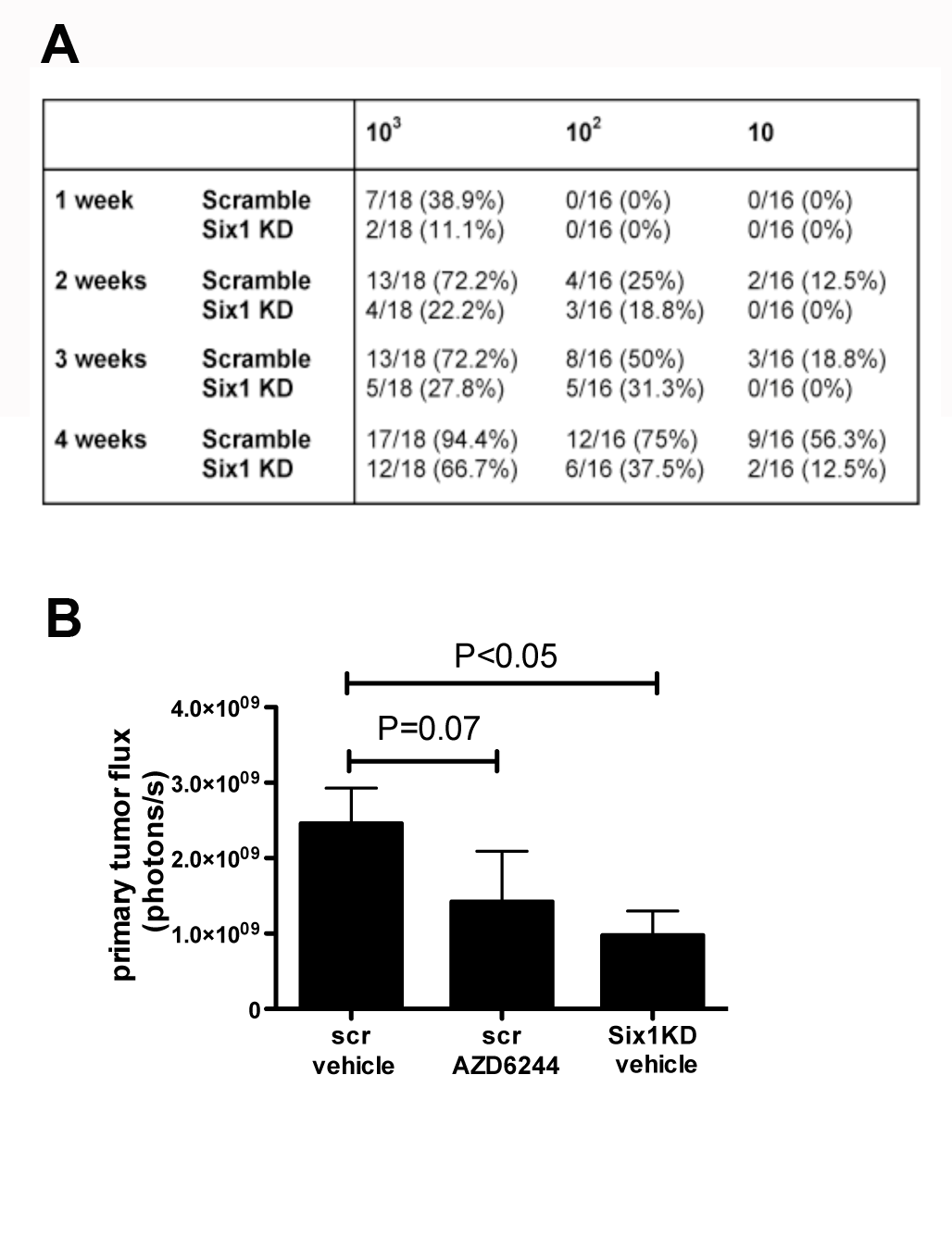

Supplement: Additional file 6 — Figure S6. Six1 KD inhibits both tumor initiation and metastasis in an immunocompetent model, and the MEK1/2 inhibitor, AZD6244, inhibits primary tumor burden similarly to Six1 KD. (A) Knockdown of Six1 inhibits tumor initiation. 66cl4 scramble control or Six1KD cells were serially diluted and then injected into the fourth mammary fat pad of six-week old female BALB/c mice. Mice were monitored for tumor initiation weekly. The data from two, three and four weeks post injection of tumor cells are shown. (B) A total of 106 66cl4/scramble or 66cl4/Six1KD cells were injected into the fourth mammary fat pad. One week post injection, mice were treated by oral gavage with vehicle or AZD6244 at a concentration of 50 mg/kg or vehicle twice per day for seven days. After injection of 150 mg of luciferin/kg into the mice, IVIS imaging was used to quantitate the primary burden at three weeks post injection. Error bars represent mean value +/- SEM. It should be noted that the signal from a number (5/10) of the scramble control tumors was saturated at this time point, suggesting that the size of these tumors may be underestimated. In contrast, only 1/8 of 66cl4/scramble tumors in the AZD6244 group had saturated signal, and none of the tumors in the 66cl4/Six1 KD group had saturated signals. [file bcr3219-S6.TIFF]
